# Association between Dopamine D4 Receptor Gene Polymorphism and Scores on a Continuous Performance Test in Korean Children with Attention Deficit Hyperactivity Disorder

**DOI:** 10.4306/pi.2009.6.3.216

**Published:** 2009-08-03

**Authors:** Bora Kim, Min-Seong Koo, Jin-Yong Jun, Il Ho Park, Dong-Yul Oh, Keun-Ah Cheon

**Affiliations:** 1 Department of Psychiatry, Kwandong University College of Medicine, Goyang, Korea.; 2 Settlement Support Center for Dislocated North Koreans, Ministry of Unification, Anseong, Korea.

**Keywords:** Attention deficit hyperactivity disorder, Continuous performance test, Dopamine D4 receptor, 4 repeats allele, Commission errors

## Abstract

**Objective:**

The aim of this study was to evaluate the association between a variable number of tandem repeats polymorphism at the dopamine D4 receptor gene (DRD4) and the performance of children with attention deficit hyperactivity disorder (ADHD) in a continuous performance test (CPT).

**Methods:**

This study included 72 ADHD children (mean age=9.39±2.05 years) who were recruited from one child psychiatric clinic. The omission errors, commission errors, reaction time and reaction standardization in the CPT were computed. The number of 48-base pairs tandem repeats in the exon III of DRD4 was analyzed in a blind manner.

**Results:**

The homozygosity of the 4-repeat allele at DRD4 was significantly associated with fewer commission errors (t=2.364, df=28.685, p=0.025) and standard deviation of reaction time (t=2.351, df=24.648, p=0.027) even after adjusting for age. The results of analyses of CPT measured values among three groups showed that the group with higher frequency of the 4-repeat allele showed a lower mean score of commission errors (F=4.268, df=2, p=0.018).

**Conclusion:**

These results suggest a protective role of 4-repeat allele of the DRD4 polymorphisms on commission errors in the CPT in children with ADHD.

## Introduction

Attention deficit hyperactivity disorder (ADHD) is a disease with principal symptoms of inattention, hyperactivity and impulsivity. The results of various epidemiological studies report a prevalence rate of 8-12%. This disease is one of the representative childhood and adolescent mental disorders occurring most commonly in children aged 4-11 years old,^1^ and tends to develop before the age of 7. About 85% of patients continue to experience symptoms to adolescence,^2^ along with a drop in academic performance and difficulty in social adaptation. Since this disorder may develop into various behavioral problems and secondary emotional disturbances, such as a decline in selfesteem, psychologically shriveling, conduct disorder, oppositional defiant disorder, anxiety disorder or mood disorder, it is essential to detect ADHD and introduce proper treatment early.^3^

The meta-analytic results of 1) a multi-ethnicity population-targeted family study, 2) a twin study, and 3) an adoption study indicate that ADHD showed about 76% heritability, and the implication is that hereditary factors play an important role in pathogenesis.^4^,^5^ Recently, genes that may be correlated with ADHD's neurobiological base have been revealed through candidate gene studies. Dopamine D4 receptor Gene (DRD4), located in the chromosome 11 (11p15.5), among them, is a specific area of attention. The 7-repeat allele of the exon III variable number of tandem repeats (VNTR) polymorphism of the DRD4 gene resulted in a blunted response toward dopamine.^6^ In a study of correlation between ADHD and DRD4 gene, the meta-analytic results of case-control studies and family-based studies demonstrated significant pooled odds ratios of 1.9 (95% confidence interval of 1.4-2.2) and 1.4 (95% confidence interval of 1.1-1.6), respectively.^7^ A group of Korean children of alcoholics were significantly more likely to carry the 4-repeat allele and the 4/4 genotype of DRD4 than control subjects, indicating the possibility of genetic vulnerability toward alcoholrelated disorders along with high morbidity to ADHD and the possibility of unfavorable therapeutic responses.^8^ In a study targeting adolescent girls in Korea, the most common type of DRD4 polymorphism was shown to be the 4-repeat allele. Unlike European or Caucasian subjects, Asians such as Koreans, Japanese and Chinese are known to rarely exhibit the 7-repeat allele of DRD4 exon III 48-bp VNTR polymorphism in exon III of the DRD4 gene.^9^,^10^ Accordingly, this study examines the distribution of DRD4 gene polymorphism and correlation with treatment responce in ADHD children in Korea.

## Methods

### Subjects

The subject group in this study included 72 male children with aged 6 to 15 years old, who visited the Outpatient Clinic of the Department of Pediatric and Adolescent Psychiatry at the Kwandong University College of Medicine Myongji Hospital. The subjects had been diagnosed as having ADHD by a psychiatrist specialized in children and adolescents. Diagnosis of ADHD was based on Diagnostic and Statistical Manual of Mental Disorders, Fourth Edition (DSM-IV) diagnostic criteria. The subjects were in drug-naïve state. The consent for the study was obtained from children's parents or representatives. Exclusion criteria of subjects were 1) current cases with a convulsive disorder and other neurologic defect including brain damage, 2) schizophrenia and cases with a history of psychosis, 3) cases with developmental problems such as autism and learning disability, and 4) those who did not provide consent to participate in this study. This study was approved by the Institutional Review Board (IRB).

### Diagnostic and evaluation tools

#### Korean Kiddie-Schedule for Affective Disorders and Schizophrenia-Present and Lifetime Version

Korean Kiddie-Schedule for Affective Disorders and Schizophrenia-Present and Lifetime Version is a semi-structured clinical interview designed to assess the present, life-time morbidity state and severity of ADHD of 32 psychiatric diseases of children and adolescence, based on the DSM-IV diagnostic criteria.^11^ In Korea, translated by Kim et al.^12^ the reliability and validity of ADHD, tic disorder, oppositional defiant disorder, depressive disorder and anxiety disorder were studied. Assessment targeting parents and children was carried out by pediatric and adolescent psychiatrists.

#### Korean Attention Deficit Hyperactivity Disorder Rating Scale-IV

In order to assess the severity of ADHD symptoms of school-aged children, the Korean ADHD Rating Scale-IV (K-ARS) was designed based on the DSM-IV diagnostic criteria consisting of 18 items.^13^ The Korean version development and standards were set up and the content was divided into odd-numbered items which reflected the symptoms of inattention and even-numbered items that reflected the symptoms of hyperactivity and impulsivity, having nine items in each category to measure the total score.^14^ Either parents or a teacher was able to mark the scale. The K-ARS, completed by parents, was assessed in this study.

#### Continuous Performance Test

ADHD is known to be associated with disorders of executive and frontal lobe function.^15^,^16^ Various neuropsychological assessments have been developed in order to measure these functional disorders objectively. CPT, among them, may be used to assess inattention, impulsivity and hyperactivity quantitatively and is largely used for ADHD diagnosis and its therapeutic assessment.^17^

The CPT presents stimuli-target and non-target in a mixture at regular intervals. The CPT is a tool to test task vigilance and distractibility by allowing the subject to react in the presence of a prescribed specific target. Various types of stimuli, such as letters, shapes, numbers and sounds are used. The CPT types are diverse depending on the form of stimuli.^18^

The form of CPT used in this study is a Korean language version developed and standardized by Shin et al.^19^ It is a clinical version of the ADHD diagnostic system (ADS). With a computerized CPT program, attention may be assessed by visual and auditory stimuli, and all results are automatically scored. Subjects are asked to practice before undertaking this test. In this study, visual stimuli incorporating shapes were given. The test duration of children 1) age 6-7 years old was ten minutes, and 2) children at an age of seven years would require fifteen minutes.

In this study, in the presence of a target stimulus, four indices were as follows:

1) Number of cases where a response is missed-namely, omission error, as an indicator of inattention; 2) Number of cases where a response occurs in the presence of non-target-these are commission errors, an indicator of hyperactivity or impulsivity; 3) Mean reaction time that measures the speed of process handling as a hit response time toward target stimuli; and 4) Standard deviation of reaction time that measures vigilance.^19^

The results were converted into values of assessments on the basis of standard computation from the normal group of the same age. The measured values were used for statistical analyses.

#### Genotyping

Blood collected from the peripheral veins of subjects in this study was treated with ethylenediaminetetraacetic acid (EDTA), transferred to a tube, and then stored frozen at -70℃ before the experiment began. A Genomic DNA Extraction Kit (Bioneer, Korea) was used to perform genomic DNA extraction from the lymphocytes of 1 mL whole blood. The established Kotler et al.^20^ method was used to perform DRD4 genotyping.

The sequence and detailed analytic method of oligonucleotide primers used to form DRD4 exon III polymorphic region (2-10 variable repeat units, 1 unit=48 bp) were as follows:

Primers: (5'-ACCACCACCGGCAGGACCCTCATGGCCTTGCGCTC-3' and 5'-CTTCCTACCCTGCCCGCTCATGCTGCTGCTCTACTGG-3') Amplification of polymerase chain reaction (PCR) was carried out in a 20 µL solution containing 100 ng genomic DNA, 10 pmol of each primer, 1 X PFU PCR buffer (Solgent, Korea), 400 µM dATP, dTTP, dCTP, 200 µM GTP (Solgent, Korea), 200 µM 7-Deaza-dGTP (Boehringer Mannheim) and 5% DMSO, 2 U Solgent PFU Taq.

With respect to PCR response, denaturation occurred at 98℃ for five minutes, and the three-phase extension process at 98℃ for 45 seconds, 55℃ for 45 seconds and 72℃ for 1 minute and 30 seconds was carried out for 35 cycles. Then, the final extension was performed at 72℃ for 5 minutes. The PTC-100 thermal cycler (MJ research, MA, USA) was used for thermal cycling, and PCR products were verified by bands seen through the ultraviolet (UV) transilluminator via ethidium bromide staining after carrying out electrophoresis using a 2% agarose gel.

#### Statistical Analyses

With respect to statistical analysis, the target group was divided by the presence or absence of the 4-repeat allele of DRD4, and an independent t-test was used to make comparative analysis of differences in scores of CPT. Also, one-way analysis of variance (ANOVA) was used to analyze the correlation between the 4-repeat allele and CPT measured values by dividing them into 3 groups-Group with no 4-repeat allele, Group with one 4-repeat allele and Group with both 4-repeat allele. The Statistical Package for the Social Science (SPSS; SPSS Inc., Chicago, IL, USA) for Windows was used for all statistical analyses. Values less than 0.05 were defined as being sta-tistically significant.

## Results

### Demographic and clinical characteristics

The 72 male ADHD children were assessed (9.39±2.05 years). The average intelligence quotient was 105.12±16.33, and the mean K-ARS score was 33.02±8.19. The distribution of ADHD diagnostic subtypes, diagnosed by K-SADS-PL-K, showed combined type, 48.6%; inattentive type, 41.7%; and hyperactive-impulsive type, 9.7%. Comorbidity shown in 38.9% of the subjects and revealed, in decreasing order, mood disorder, 22.3%; anxiety disorder, 12.5%; tic disorder, 9.7%; and conduct disorder and oppositional defiant disorder, at 2.8% each. Comorbidity of having two or more disorders included a total of 7 cases: 3 cases of mood disorder+anxiety disorder, 1 case of mood disorder+conduct disorder, 1 case of anxiety disorder+oppositional defiant disorder, 1 case of mood disorder+oppositional defiant disorder, and 1 case of mood disorder+anxiety disorder+tic disorder (Table 1).

### Variable number of tandem repeat polymorphism of dopamine D4 receptor gene

Among the 144 chromosomes of 72 samples in this study, the 4-repeat allele (80.5%) showed the highest frequency among exon III 48-bp VNTR polymorphism of DRD4, and the 2-repeat allele (14.6%) showed the next highest frequency. The 5-repeat allele and 6-repeat allele showed distributions of 2.1%, 2.8% each. The 7-repeat allele was not shown. The genetic distribution of exon III VNTR polymorphism of DRD4 coincided with the expected values of the Hardy-Weinberg Equilibrium (goodness of fit χ^2^=1.13, df=5, p=0.89).

Among the genotypes of DRD4, the 4/4 genotype was observed in 49 (68.1%) out of 72 samples, the 2/4 genotype in 13 (18.1%), the 2/2 and 4/6 genotype in three (4.2%) each. The 4/5 genotype was observed in two (2.8%) and the 2/5 and 2/6 genotype in one (1.4%) each (Table 2).

### Association between the genotype at dopamine D4 receptor and scores of Continuous Performance Test

The results of comparative analyses of scores for commission error and standard deviation of reaction time in CPT, depending on homozygosity of 4-repeat allele of DRD4 genes, identified a significant difference in the mean score of these two groups (Table 3). The group carrying homozygosity of the 4-repeat allele tended to have lower scores of commission errors (t=2.364, df=28.685, p=0.025) and standard deviation of reaction time (t=2.351, df=24.648, p=0.027) in CPT.

Performance on the CPT was analyzed by subdividing into three groups-4/4 genotype group having two 4-repeat alleles; 2/4, 4/5 and 4/6 genotype having only one 4-repeat allele; 2/2, 2/5 and 2/6 genotype having no 4-repeat allele. Subjects having greater instance of the 4-repeat allele showed a significantly lower mean score for commission errors (F=4.268, df=2, p=0.018) (Table 4).

## Discussion

The results of the present study identified a significant association between homozygosity for the 4-repeat allele at the DRD4 gene and lower scores of commission errors and standard deviation of reaction time in CPT. These findings suggest that the 4-repeat allele of DRD4 gene plays a protective role against inattention and impulsivity of ADHD symptoms.^21^,^22^ This is consistent with previous work^23^ in which higher scores of commission errors in CPT had been measured in ADHD children with 7-repeat allele of DRD4, while lower omission errors and commission errors had been measured in patients with homozygosity of the 4-repeat allele of DRD4 genes. Barr et al.^24^ found, using the transmission disequilibrium test (TDT) that the results of dominant transmission of 7-repeat allele and non-transmission of 4-repeat allele were significant. They also mentioned the possibility of a protective role of 4-repeat allele against ADHD development. In line with the results of that investigation, a study of correlation between the VNTR polymorphism in exon III of DRD4 gene and the therapeutic results of methylphenidate conducted in Korean ADHD children reported that the group with a good treatment response of 50% or more symptomatic improvement had significantly higher values of 4/4 genotype of DRD4 than did the group with a poor treatment response of less than 50% symptomatic improvement.^25^ Previous work targeting on Asian population^9^,^10^ reported that almost no incidence of 7-repeat allele was found in VNTR polymorphism in exon III of DRD4. This study also found a high distribution of the 2-repeat allele (14.6%), which was lower in frequency than the 4-repeat allele, and the 2/4 genotype was verified in 13 out of 72 (18.1%) subjects. A recent study targeting Chinese ADHD children reported that the frequency of the 2-repeat allele patient group was higher than that of the normal control group.^26^ This investigation also made a comparative analysis of CPT measured values of 4/4 genotype and 2/4 genotype in order to verify the differences of inattention, hyperactivities and impulsivity between 4-repeat allele and 2-repeat allele. The measured value of commission errors (t=-2.309, df=15.946, p=0.035), the standard deviation of mean reaction time (t=-2.365, df=12.660, p=0.035) of the group with 2/4 genotype was higher than that with 4/4 genotype. Therefore, it would be meaningful in the future study of ADHD and 2-repeat allele to inquire into differences in the endophenotype in accordance with polymorphism of 2-repeat allele by securing a target group with a larger number of subjects.

The limitations of this study are as follows: First, the number of subjects was relatively small. This study involved target patients visiting the outpatient clinic of a university hospital in a specific area and may thus offer limited generalization of the conclusions. Second, comorbidity and psychosocial variables that may affect CPT results have not been controlled. In this study, 28 out of 72 subjects (38.9%) were verified as having comorbid disorders. ADHD has a high frequency of comorbidity of approximately 50% or more, and other psychiatric disorders such as anxiety disorder and mood disorder may also include the symptom of "inattention." Comorbid tic disorder seen concurrently in 40-60% of ADHD cases may largely affect the characteristics of the target group. Hence, future studies should be conducted controlling such an element. Lastly, it is not clear whether or not the CPT used in this study is a sensitive test that to detect inattention of ADHD, hyperactivity and impulsivity. Taken together, previous work demonstrates more commission errors and omission errors as well as differences in the standard deviation of reaction time, sensitivity and reaction time in CPT with ADHD patients as opposed to normal children.^17^,^27^,^28^ Nevertheless, the inattention, hyperactivity and impulsivity seen in ADHD are complex phenomena arising in various areas of the brain. On the contrary, visual stimulus incorporated in CPT has been simplified and results from older or highly intelligent subjects suggest some issues of low discrimination.^29^,^30^ Despite debates on usefulness of CPT in ADHD, CPT shows somewhat consistent results as compared with other objective neuropsychological test tools. CPT is widely used in the diagnosis, treatment and research of ADHD owing to facts corroborative of reliability and validity through standardization.

However, as mentioned above, with low difficulty of CPT comes to the likelihood that results can vary depending on age and intelligence. In such cases, auditory stimuli may have a higher degree of difficulty as opposed to visual stimuli. It has been reported that, in cases with an old age or with high intelligence, auditory stimulus-incorporated CPT may be more useful in ADHD diagnosis.^19^ Thus, in the future, a study design using CPT, and targets such as visual, auditory or both stimuli would be necessary.

Notwithstanding such limitations, this investigation offers comparative analysis of CPT measured values depending on the existence of homozygosity of 4-repeat allele of DRD4 targeting on an Asian population, and provides an analysis of correlation of genetic polymorphism and CPT measured values in accordance with number of 4-repeat alleles.

In future studies, more samples from various Asian ethnic groups should be collected. Comorbidity and other psychosocial elements that might affect ADHD should be maximally controlled.

## Figures and Tables

**TABLE 1:**
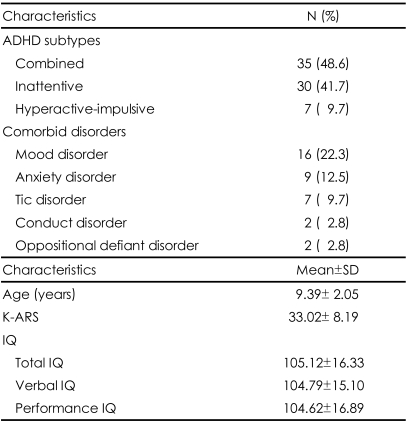
Clinical characteristics of the ADHD subjects (N=72)

N: number of subjects, SD: standard deviation, IQ: intelligence quotient, K-ARS: Korean ADHD Rating Scale

**TABLE 2:**
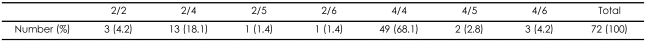
The genetic polymorphism of the DRD4 gene

By Hardy-Weinberg equilibrium (goodness of fit χ^2^=1.13, df=5, p=0.89). DRD4: dopamine D4 receptor

**TABLE 3:**
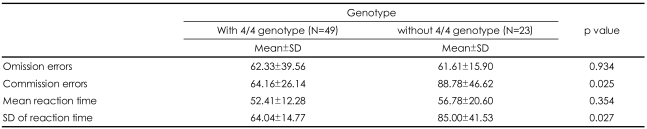
Comparison of the scores in Visual Continuous Performance Test between the ADHD children with and without the 4/4 genotype at DRD4

N: number of subjects, SD: standard deviation, with/without 4/4 genotype: ADHD children with/without homozygosity for the 4-repeat allele at DRD4, DRD4: dopamine D4 receptor

**TABLE 4:**
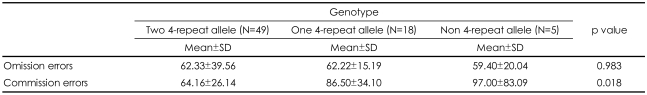
Correlation between the scores in the Visual Continuous Performance Test and the number of DRD4 4-repeat alleles

N: number of subjects, SD: standard deviation, DRD4: dopamine D4 receptor gene
